# Multi-omics analysis reveals metabolic regulation of phosphatidylcholine, triglycerides, phosphatidylethanolamine, and cardiolipin metabolism in mouse liver with metabolic dysfunction-associated steatotic liver disease

**DOI:** 10.1371/journal.pone.0332177

**Published:** 2025-11-07

**Authors:** Hong Liang, Kang Song

**Affiliations:** 1 Department of Basic Medical Sciences, Medical College, Qinghai University, Xining, Qinghai, China; 2 Endocrinology Department, Qinghai Provincial People’s Hospital, Xining, Qinghai, China; Al-Azhar University / King Khalid University, EGYPT

## Abstract

The dysregulation of phosphatidylcholine (PC), triglycerides (TG), phosphatidylethanolamine (PE), and cardiolipin (CL) metabolism is believed to contribute to the development of MASLD. However, little is known about the mechanisms underlying the onset of this condition. To establish a mouse model of MASLD, C57BL/6J mice were fed a high-fat diet (HFD). Lipidomics was applied to identify differences in liver lipids. RNA-sequencing and bioinformatics analyses were conducted to investigate changes in the expression of genes and pathways associated with these metabolic processes. 49 lipid classes and 3221 lipid species were identified using positive- and negative-ion pattern identification. A total of 678 differentially expressed genes were identified, of which 364 were upregulated and 314 were downregulated in the MASLD group. KEGG enrichment pathway analysis highlighted the downregulation of four genes such as *Gpat4*, *Gpcpd1*, *Chkb*, and *Etnppl.* These findings contribute to our understanding of the metabolic changes associated with MASLD.

## 1. Introduction

Metabolic-dysfunction-associated steatotic liver disease (MASLD), also known as non-alcoholic fatty liver disease, affects approximately one-quarter of the global adult population and poses significant global health and economic burdens [1–3]. A meta-analysis involving data from more than 13 million individuals in Asia indicated a 29.6% prevalence of MASLD [4]. This high prevalence is associated with poor lifestyle habits including prolonged sitting, low levels of physical activity, and high-calorie food intake.MASLD diagnosis is based on the identification of hepatic steatosis and presence of at least one of three metabolic conditions: that is, overweight/obesity, type 2 diabetes (T2DM), or metabolic dysfunction [5].

Metabolic disorders are important contributors to the genesis and development of MASLD [6]. In MASLD, during the process of liver fat accumulation, inflammation, fibrosis, and cancer can be aggravated owing to intracellular damage and liver insulin resistance [7]. MASLD increases the risk of not only liver-related complications, but also several extrahepatic diseases [5]. The risk of developing new T2DM is approximately 2 times higher in people with MASLD compared with that in people without MASLD [8]. MASLD is closely related to systemic energy metabolism disorders, with atherosclerotic dyslipidemia being one of them [9]. Moreover, MASLD has been reported to be a risk factor for chronic kidney disease (CKD) [10]. Metabolic disorders are among the potential mechanisms associating MASLD with CKD [11].

The liver plays an important role in regulating lipid homeostasis.The pathological accumulation of triglycerides and other lipids in hepatocytes is characteristic of MASLD [9]. The first step in the development of MASLD involves the accumulation of triglycerides (TG), a type of glycerolipid, in liver lipid droplets. Glycerophospholipids, including phosphatidylcholine (PC), phosphatidylethanolamine (PE), and cardiolipin (CL), serve as important structural components of cell membranes [12]. Imbalances in the metabolism of glycerophospholipids may negatively affect membrane stability and promote the development of MASLD [13].

Lipidomics, using high-throughput analytical techniques, enables the comprehensive evaluation of changes in the composition and levels of lipids in organisms. A lipidomic analysis enables the assessment of biological processes related to lipid families and molecules, highlighting their functions [14]. Several recent lipidomic studies have revealed significant changes in fatty acid patterns and phospholipid composition in liver samples from patients with MASLD, suggesting that these conditions may be linked to lipid metabolism [15,16]. A transcriptomic study has also confirmed that these differences are mainly associated with lipid metabolism [17]. The liver tissues of patients with MASLD and healthy participants have further been analyzed using transcriptomics analysis to identify differentially expressed genes (DEGs) [18], revealing genetic signatures of steatohepatitis and fibrosis. However, few studies have explored the specific changes in the metabolism of PC, TG, PE, and CL in MASLD.

To address this issue, in the present study we aimed to elucidate MASLD-associated changes in the metabolic profiles of PC, TG, PE, and CL using lipidomic analysis in high-fat diet (HFD)-fed mice. Moreover, we aimed to identify changes in the pathways and genes associated with liver glycerolipid and glycerophospholipid metabolism in MASLD using integrated transcriptomics. Our findings provide important insights regarding the changes in liver metabolism in MASLD.

## 2. Materials and methods

### 2.1. Animals

Twenty male C57BL/6J mice aged six weeks were purchased from Chengdu Dashuo Experimental Animal Co., Ltd. (Chengdu, China). After one week of adaptive feeding, mice were divided randomly and equally into two groups as follows (n = 10 each): (1) mice fed a normal diet (Chengdu Dossy Experimental Animal Co., Ltd.) as the control group and (2) those fed HFD (D12492, Research Diets, New Brunswick, NJ, USA) as the MASLD group. The feeding period was 12 weeks, and mice were weighed every 4 weeks. Specific pathogen-free conditions were maintained with a humidity of 55 ± 10%, a 12 h dark-light cycle, and unrestricted feeding and watering.

**Fig 1:**
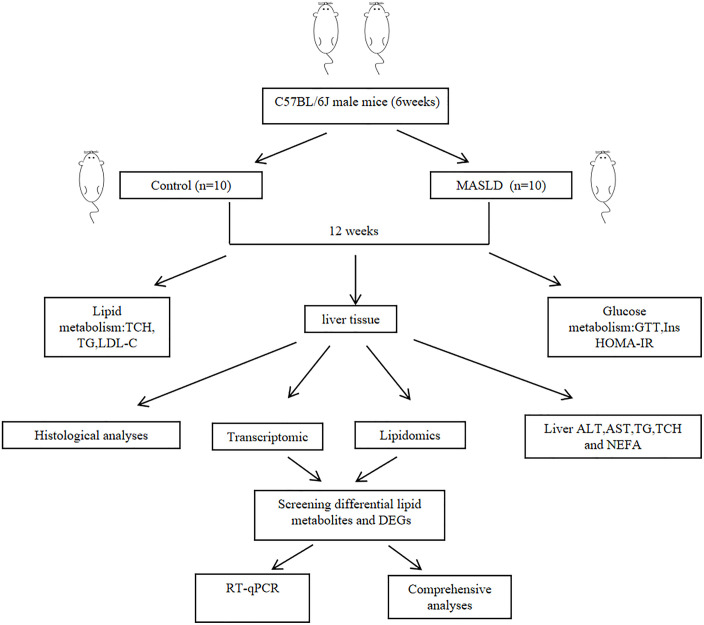
Study process Flowchart.

The study protocol was approved by the Ethics Committee of Qinghai Provincial People’s Hospital (approval number 2021−37). All animal experiments were conducted in accordance with the Animal Control Regulations of the Ministry of Health, China.The study design is shown in Fig 1.

After 12 weeks of feeding, all mice were euthanized by intraperitoneal injection of pentobarbital sodium (50 mg/Kg; Sigma-Aldrich, St. Louis, MO, USA).

### 2.2. Glucose tolerance test

Following intraperitoneal injection of 1.5 g/kg glucose after 8 h fasting, blood glucose levels were measured at 0, 15, 30, 60, and 120 min. Blood glucose measurements were performed using a standard blood glucose meter (Xinjiang Hengsheng Biological Co. Ltd., Urumchi, China) after collecting tail venous blood samples.

### 2.3. Liver samples

Liver tissues were collected and weighted. Liver tissues were fixed in 4% (w/v) paraformaldehyde for morphological assessment. The remaining tissue was fixed in liquid nitrogen and stored at −80°C. Frozen sections were prepared and used for lipidomics and transcriptomics analyses.

### 2.4. Blood biochemistry

Total cholesterol (TCH), TG, and low-density lipoprotein cholesterol levels were determined using a biochemical analyzer for veterinary use (Shenzhen Mindray Biomedical Electronics Co., Ltd., Shenzhen, China). Insulin levels were determined using an enzyme-linked immunosorbent assay kit (ZC-38920; Shanghai Zhuocai Biotechnology Co., Ltd., Shanghai, China). Based on the homeostatic model assessment of insulin resistance (HOMA-IR), glucose concentrations were calculated as follows:

glucose concentration (mmol/L) × insulin concentration (mU/mL)/22.5.

### 2.5. Liver TG, TCH, alanine transaminase (ALT), glutamic-oxalacetic transaminase (AST), and non-esterified fatty acid (NEFA) levels

The levels of TG, TCH, ALT, AST and NEFA in liver tissues were determined using commercial kits (A110-1–1, A111-2–1, C009-2–1, C010-2–1 and A042-1, respectively; Nanjing Jiancheng Bioengineering Institute, Nanjing, China) according to the manufacturer’s instructions.

### 2.6. Hematoxylin and eosin (H&E) staining

Liver tissues were fixed with 4% (w/v) paraformaldehyde, dehydrated using an ethanol gradient, and embedded in liquid paraffin for preparation of sections (2–4 μm thickness). Sections were dried for 5 min in an oven, dewaxed, dehydrated, and stained with H&E. A digital slice scanner (Panoramic 250, 3DHISTECH, Budapest, Hungary) was used for image collection. Images were captured at 100 × magnification and 400 × magnification.Each experimental group comprised three biological replicates. Hepatocyte lipid droplets were quantified in liver tissue sections from each sample, with ten measurements recorded per section. Subsequent statistical analysis was performed on the mean values derived from the three replicates.

### 2.7. Oil red O staining

Frozen tissues at −80°C were sectioned to approximately 2 mm, embedded, sectioned, and stained with Oil red O (YO7512, Hefei Bomei Biotechnology Co., Ltd., Hefei, China). In the acquired images, the area of lipid droplets was measured using the Image-Pro Plus 6.0 image analysis system (Media Cybernetics, Inc., Rockville, MD, USA), with the lipid droplet expression area being calculated as follows:

lipid droplet expression area/visual field area × 100%.

### 2.8. Lipidomic analysis

#### 2.8.1. Lipid extraction.

Lipids were extracted from samples using methyl tert-butyl ether (MTBE).Liver tissue (30 mg) was combined with 20 μL lipid standards, and was homogenized twice (1 min each time) with 200 μL of water and 240 μL of methanol using an MP Fastprep-24 Automated Homogenizer (24 × 2, 6.0M/S).Subsequently, 800 μL of MTBE was added to the mixture, followed by sonication for 20 min at 4°C.The sample was left on the bench for 30 min at room temperature.After centrifugation at 14,000 × *g* for 15 min at 10°C,the top layer was gathered and dried with nitrogen.The lipid extracts were dissolved in 200 μL of a 90% isopropanol/acetonitrile mixture and centrifuged at 14,000 × *g* for 15 min. Then, 3 μL of each sample was injected into the column.Quality control (QC) samples were prepared from 10 μL aliquots of each sample. QC samples were column-adjusted at the beginning of the sample queue and then at every ten injections to evaluate differences in retention time and ion intensity over time.

#### 2.8.2. Mass spectrometry.

Spectrometric analyses were performed using an ultra-high performance liquid chromatography system (Nexera LC-30A, Shimadzu, Japan) coupled with a Q-Exactive Plus mass spectrometer (Thermo Scientific).

2.8.2.1 HPLC system: Analytes were separated on a reverse-phase charged surface hybrid C18 column (1.7 μm, 2.1 mm × 100 mm, Waters). Elution was carried out using a system consisting of solvent A (acetonitrile–water [6:4, vol/vol], 0.1% formic acid, and 0.1 mM ammonium formate) and solvent B (acetonitrile–isopropanol [1:9, vol/vol], 0.1% formic acid, and 0.1 mM ammonium formate). Lipids were eluted using a linear gradient from 30% to 100% solvent B over 23 min, followed by equilibration in 5% solvent B for 10 minutes. Fragmentation was performed in positive and negative modes. The following parameters were used: source temperature, 300 °C; capillary temperature, 350 °C; ion spray voltage, 3000 V; S-lens radio frequency (RF) level, 50%; scan range, 200–1800 m/z.

2.8.2.2 MS/MS analysis: Positive and negative ions were detected by ESI, as described previously [19]. The following parameters were used: heater temperature set to 300°C, sheath gas flow rate at 45 arb, auxiliary gas flow rate at 15 arb, sweep gas flow rate at 1 arb, spray voltage at 3.0 kV, capillary temperature at 350°C, and S-lens RF level at 50%, MS^1^ scan range: 200–1800 m/z. The mass charge ratio of lipid molecules and lipid fragments is obtained as follows: ten fragments (MS^2^ scan, Higher Energy Collision-induced Dissociation) were obtained following each complete scan.The resolution at m/z 200 was 70,000 for MS^1^ scans and 17,500 for MS^2^ scans.

2.8.2.3 Data processing and statistical analysis: LipidSearch software version 4.2 (Thermo Scientific) was used to process raw data, identify and align peaks, correct retention times, obtain peak areas, and identify lipid species and standards. The primary parameters were the following: precursor tolerance, 5 ppm; fragment ion tolerance, 5 ppm; product ion threshold, 5%. Data on over 30 lipid classes and more than 1,700,000 ion fragments were analyzed. Ammonium and acetate adducts were detected in positive and negative ion modes, respectively, because ammonium acetate was used in the mobile phase. Peaks with more than 50% missing values were excluded. After processing, data were normalized and integrated using the Pareto chart and then imported into SIMPCA-P 16.1 (Umetrics, Umea, Sweden) for multivariate analysis, including principal component analysis (PCA), partial least squares discriminant analysis (PLS-DA), and orthogonal partial least squares discriminant analysis (OPLS-DA). In OPLS-DA, lipids with variable importance in the projection (VIP) >1 and p < 0.05 were selected

### 2.9. Transcriptomic analysis

Transcriptomic analysis was carried out by Shanghai Applied Protein Technology Co., Ltd. (Shanghai, China). The liver tissues of each mouse in the control and MASLD groups were analyzed. Briefly, liver RNA was extracted using TRIzol Reagent (Sigma-Aldrich). An Agilent Bioanalyzer 4150 (Agilent Technologies, Santa Clara, CA, USA) was used to determine RNA integrity number, and qualified RNA was used to construct libraries. After the enrichment of mRNA using oligo (dT) magnetic beads, RNA was randomly fragmented using a fragmentation buffer. First-strand cDNA was synthesized using random hexamers with mRNA as a template, and second-strand cDNA was synthesized using dNTPs and DNA polymerase I. Double-stranded cDNA was purified using AMPure XP beads and subjected to end repair, A-tailing, and ligation of the sequencing adapter, with the fragment size selected using AMPure XP beads. A cDNA library was generated using PCR enrichment. The original image data file obtained from high-throughput sequencing was analyzed using CASAVA Base Calling Clean Reads and compared with the reference genome using HISAT2 software (http://daehwankimlab.github.io/hisat2/) to obtain mapped reads. Values in fragments per kilobase of transcript per million mapped reads for gene expression in each sample were calculated using featureCounts software. Differences in gene expression between the two groups were analyzed using DESeq2 (http://bioconductor.org/packages/release/bioc/html/DESeq2.html) with screening criteria |log2 fold-change (FC)| > 1 and *P*adj < 0.05. Functional enrichment of DEGs was evaluated using Gene Ontology (GO) and Kyoto Encyclopedia of Genes and Genome (KEGG) pathway enrichment analysis and the functional clusters of differential genes between the two groups were described.

### 2.10. Reverse transcription quantitative polymerase chain reaction (RT-qPCR)

The extraction of total RNA and the synthesis of cDNA were performed as previously described [19]. Primers for RT-qPCR detection are shown in S1 Table in S1 File.

### 2.11. Differentially expressed lipid metabolites and DEG correlation analysis

Spearman correlation analysis method was used to calculate the correlation coefficient between the selected significantly DEGs and significantly differentially expressed lipids in the samples. Cytoscape software (Version 3.8.2) was utilized for correlation network analysis.

### 2.12. Statistical analysis

Statistical analysis was performed using GraphPad Prism 8.0 software (GraphPad Software, San Diego, CA, USA). Quantitative data are expressed as the mean ± standard deviation. The independent sample *t-*test was used for comparison of the two groups. Significant difference was set at *P* < 0.05.

## 3. Results

### 3.1. HFD effects on body weight and liver function

**Fig 2:**
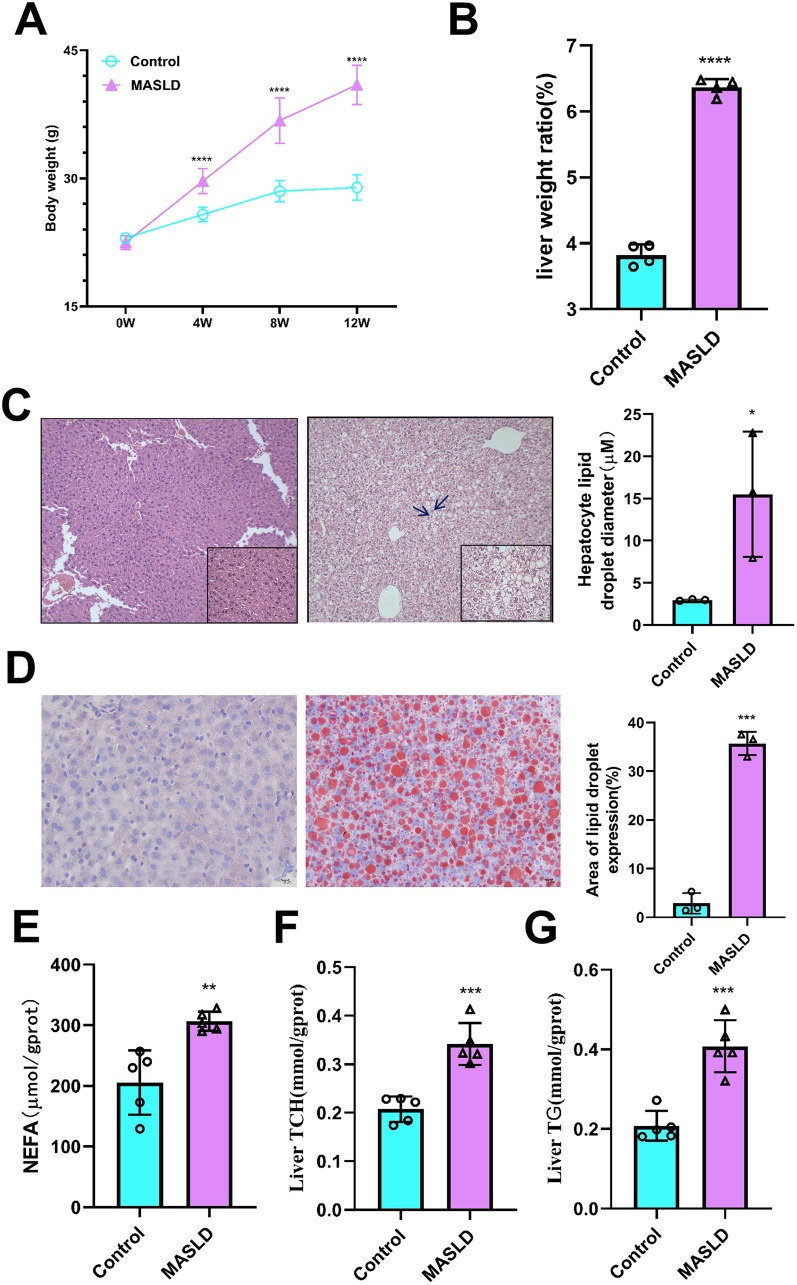
Effect of high-fat diet on body weight and liver. (A) Body weight. (B) Liver weight ratio. (C) Control and MASLD group H&E staining.The blue arrows in the figure indicate lipid droplets in the cytoplasm of liver cells. (D) Control and MASLD group Oil red-O staining, shown as percentage of lipid droplets in liver tissue. (E–G) The content of NEFA, TCH and TG in liver tissue. Data are presented as mean ± SD ((A), n = 10 per group; (B), n = 4 per group; (C) and (D), n = 3 per group; (E), (F), and (G), n = 5 per group). (A) – (D) *P < 0.05,**P < 0.01, ***P < 0.001, ****P < 0.0001.

To investigate the effects of HFD feeding on body weight and liver function, we maintained C57BL/6J mice on HFD for 12 weeks. Mice in the MASLD group exhibited significantly higher body weight, with an average weight of more than 40 g at 12 weeks, than that in the control group (Fig 2A). HFD also affected liver tissues; compared to the effects in the control mice, HFD-fed mice exhibited a higher liver weight ratio at 12 weeks (Fig 2B). Moreover, the hepatocyte cytoplasm of liver tissues collected from the MASLD group exhibited higher deposition of lipids and hepatocyte degeneration, as evidenced by H&E and Oil red O staining (*P* < 0.05; Fig 2C, D). The levels of NEFAs, TCH, and TG in liver tissue were significantly higher in the MASLD group than in the control group (*P* < 0.05; Fig 2E–G). HFD feeding affected liver function, as the levels of ALT and AST in liver tissues were significantly higher in the MASLD group than in the control group (*P* < 0.05; S1 and S2 Figs in S1 File). Collectively, these results suggest that HFD feeding affected the body weight and liver function in mice, leading to the development of MASLD.

### 3.2. HFD effects on glucose and lipid metabolism

**Fig 3:**
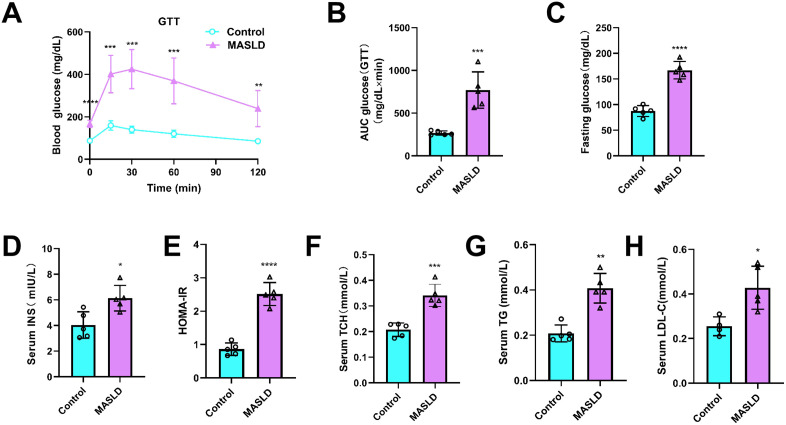
Effect of high-fat diet on glucose metabolism and lipid metabolism. (A) Glucose tolerance test. (B) Area under the curve for GTT. (C) Fasting glucose. (D) Serum insulin. (E) HOMA-IR. (F) Serum total cholesterol (TCH). (G) Serum triglyceride (TG). (H) Serum low-density lipoprotein cholesterol (LDL-C). Data are presented as mean ± SD ((A)–(E), n = 5 per group; (F)–(H), n = 4–5 per group). (A) – (H) *P < 0.05, **P < 0.01, ***P < 0.001, ****P < 0.0001.

Mice with HFD-induced MASLD exhibited significantly impaired glucose tolerance and increased fasting blood glucose concentration (*P* < 0.05; Fig 3A–C). HOMA-IR values indicated that insulin sensitivity in HFD-fed mice was also decreased compared to that in the control group (*P* < 0.05; Fig 3D, E). Moreover, HFD feeding induced dyslipidemia in MASLD mice; at 12 weeks, serum concentrations of TCH, TG, and low-density lipoprotein cholesterol were significantly higher in MASLD mice (Fig 3F–H) than in the control group. These data demonstrate metabolic abnormalities in HFD-induced MAFLD mice.

### 3.3. Analysis and comparison of lipid class and lipid species

**Fig 4:**
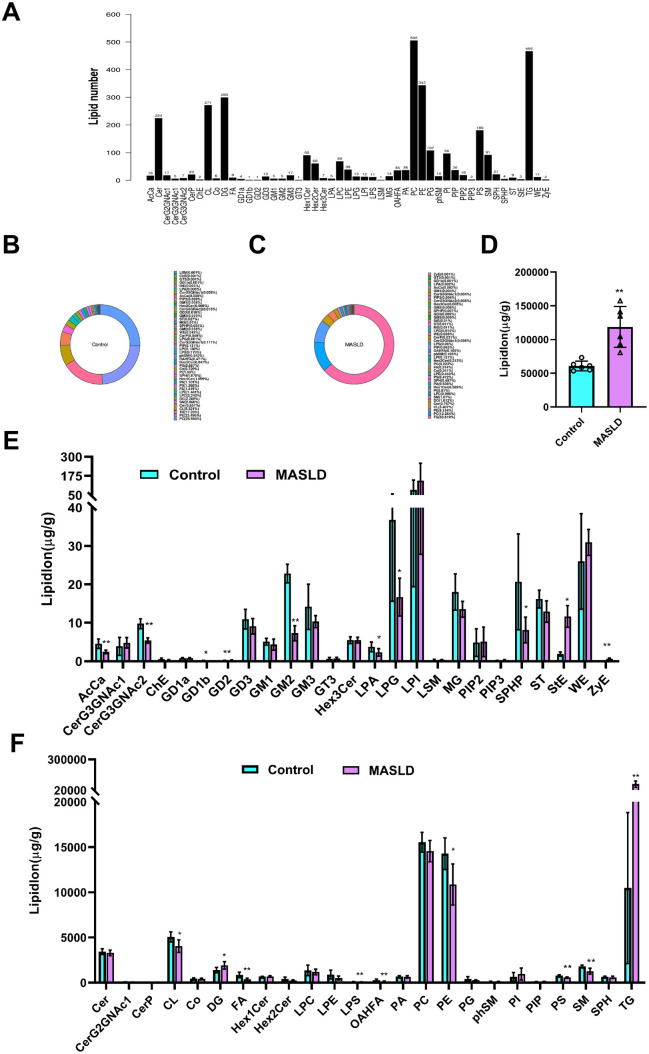
Effect of high-fat diet on hepatic lipid class and lipid species. (A) Number of lipid classes and lipid species. (B) Composition of lipid class in the control group. (C) Composition of lipid class in the MASLD group. (D) Total lipid molecule content. (E) and (F) Content of different lipid subgroups between the control and MASLD group. Data are presented as mean ± SD ((D) – (F), n = 6 per group). (D) – (F) *P < 0.05, **P < 0.01.

We identified 49 lipid classes and 3221 lipid species in the MASLD and control groups using positive- and negative-ion pattern identification. The PC, TG, PE, and CL classes comprised 505, 466, 343, and 271 lipid species, respectively (Fig 4A). In the MASLD group, TG content was the highest at 63.6%, followed by PC (12.3%), PE (9.2%), and CL (3.4%) (Fig 4C). In the control group, the PC content was the highest at 25.6%, followed by PE (23.5%), TG (17.2%), and CL (8.3%) (Fig 4B). Moreover, the levels of total liver lipids in the MASLD group increased by 95.35% compared to those in the control group (Fig 4D).

Comparison of the 49 lipid classes revealed significant differences in 19 lipid classes between the two groups (Fig 4E, F). TG and diglyceride contents were increased in the MASLD group, whereas those of the 17 other lipid classes were higher in the control group. The level of liver PC was lower in MASLD mice than in control mice. These results indicate that the levels of TG, which is involved in glycerolipid metabolism, and PE and CL, which are involved in glycerophospholipid metabolism, are altered in HFD-induced MASLD.

### 3.4. Comparative analysis of differential lipids using univariate and multivariate statistical approaches

**Fig 5:**
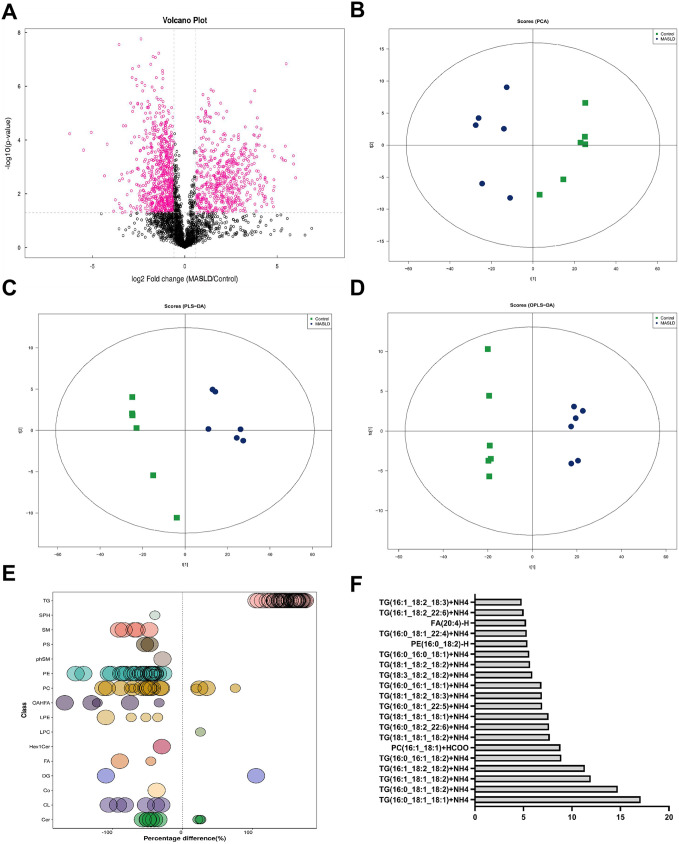
Changes in differential lipid molecules in high-fat diet-induced MASLD. (A) Volcano plot of the control and MASLD groups. Differential molecules are represented by rose-red circles, with the black circles showing the neutral lipid molecules. (B) PCA score plot. (C) PLS-DA score plot. (D) OPLS-DA score plot. (E) Bubble chart of hepatic altered lipid species in MASLD group. (F) Top 20 lipids with the highest VIP value. The abscissa represents the VIP value. Data are presented as mean ± SD ((A) – (F), n = 6 per group).

We performed univariate analyses to further clarify the changes in lipids in the HFD-induced MASLD and control groups. Fig 5A shows differentially expressed lipid molecules based on FC > 1.5 or < 0.67 with *P* < 0.05. We used principal component analysis (PCA), a multivariate analytical method, to illustrate the overall distribution trend in samples and the degree of difference between the MASLD and control groups. The PCA simulation parameter R^2^X was 0.84, indicating a stable and reliable model (Fig 5B, S2 Table in S1 File). The model evaluation parameters, R^2^Y and Q^2^ for partial least squares discrimination analysis (PLS-DA) were 0.995 and 0.935, indicating that the model was stable and reliable (Fig 5C, S3 Table in S1 File). We used a third method, orthogonal PLS-DA, to confirm the stability and reliability of the model, for which the values of R^2^Y and Q^2^ were 0.995 and 0.925, respectively (Fig 5D, S4 Table in S1 File). Fig 5E shows the differentially expressed lipid molecules in MASLD group, with glycerolipids (TG) and glycerophospholipids (PC, PE, and CL) as the main lipids. Among the 20 lipids with the highest VIP values, 17 were TG, whereas the 6^th^, 16^th^, and 18^th^ places were PC, PE, and FA, respectively (Fig 5F). A total of 154 lipid molecules with significant differences were detected, of which TG (39.61%) and PE (21.43%) were the most common.

### 3.5. Changes in TG, PC, PE, and CL in HFD-induced MASLD

**Fig 6:**
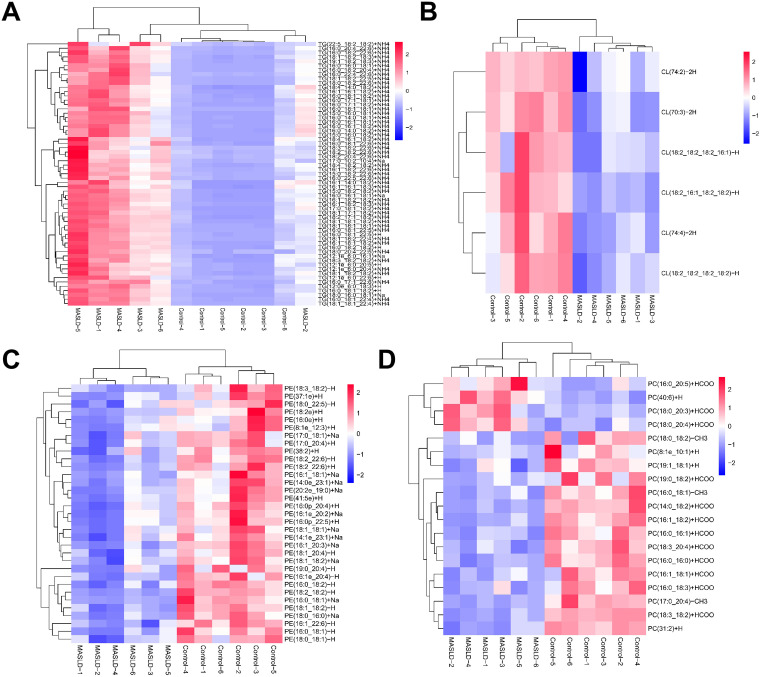
Change in glycerolipids and glycerophospholipids between in high-fat diet-induced MASLD. (A) Heatmap analysis of TG species, (B) CL species, (C) PE species, and (D) PC species. Data are presented by mean ± SD (A-D, n = 6 per group).

TG is a primary substrate for lipid oxidation. We analyzed a total of 61 TG species with significant differences using high-resolution non-targeted approaches; the detailed changes in these species are displayed in Fig 6A, S5 Table in S1 File. TG content was significantly increased in HFD-induced MASLD. TG species with chain lengths of 50–56 carbons and 2–5 bonds were present at higher levels in MASLD than in the control group.

The CL, PE, and PC glycerophospholipids were present at markedly lower levels in HFD-induced MASLD than in the control group, with total CL being significantly decreased in MASLD (Fig 6B, S5 Table in S1 File). We detected six CL species with significant differences, all showing a decline. The levels of CL (18:2_18:2_18:2_16:1)-H and CL (18:2_16:1_18:2_18:2)-H were 69% and 63% lower, respectively, in MASLD than in the control group. Moreover, we analyzed 34 PE species using lipidomic approaches; detailed changes in these species are shown in Fig 6C, S5 Table in S1 File. Consistent with the trend for total CL, all PE species were present at lower levels in the MASLD liver than in the control one. Notably, among the 19 PC species with significant differences, four species were present at higher levels (Fig 6D, S5 Table in S1 File). The levels of PC (18:0_20:3) +HCOO and PC (40:6) +H were 121% and 42% higher, respectively, in the MASLD group than in the control group, whereas levels of the other 15 PC species were significantly lower. In summary, variable decreases in major phospholipid classes were observed in the livers of mice with HFD-induced MASLD.

### 3.6. Liver transcriptomic changes in HFD-induced MASLD

**Fig 7:**
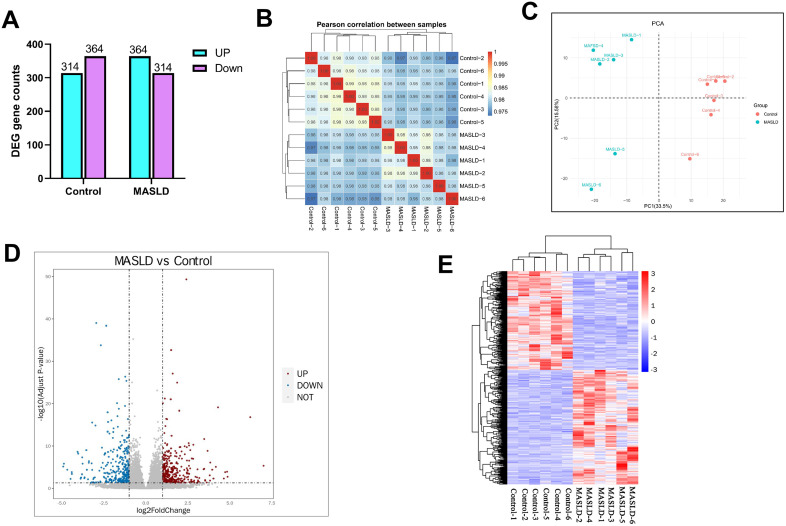
Effect of high-fat diet on liver transcriptomics. (A) Differentially expressed gene counts between control and MASLD groups. (B) Correlation analysis of gene expression patterns. (C) PCA analysis of control and MASLD groups. (D) Volcanic map of differential gene expression distribution. In comparison with the control group, gray dots represent genes with no significant differences to the MASLD group, red dots represent significantly upregulated genes, and blue dots represent significantly downregulated genes. (E) Differential gene cluster map. Data are presented as mean ± SD ((A)–(E), n = 6 per group).

We performed transcriptomic analysis (RNA-sequencing; RNA-Seq) to reveal the mechanisms underlying the regulation of lipid metabolism in HFD-induced MASLD. We identified a total of 678 DEGs (*P* < 0.05), of which 364 were upregulated and 314 were downregulated in the MASLD group (Fig 7A). To demonstrate the reliability of the test and the rationality of the biological duplication of samples, we conducted a correlation test for gene expression levels between samples. The correlation coefficients were greater than 0.97, indicating high similarity in expression patterns between samples (Fig 7B); the PCA results showed similar results (Fig 7C). A volcano plot showing the distribution and upregulation of DEGs between the MASLD and control groups is shown in Fig 7D. A heatmap confirming the changes in the expression of different genes between the two groups is shown in Fig 7E.

### 3.7. GO and KEGG enrichment analysis of DEGs in HFD-induced MASLD

**Fig 8:**
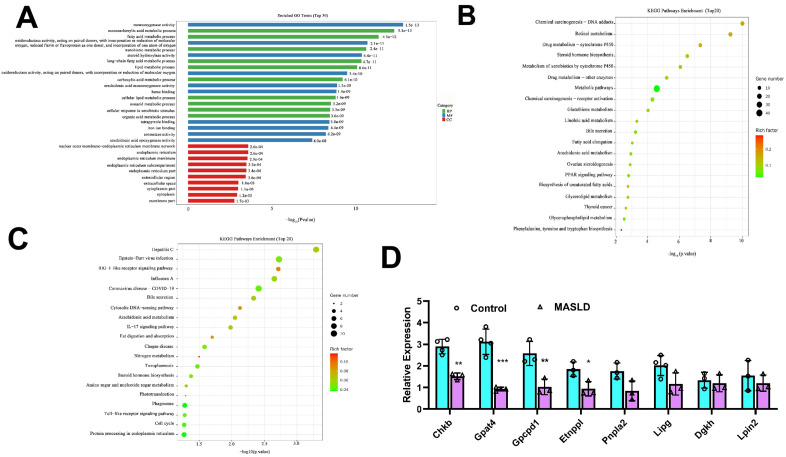
GO and KEGG pathway annotation of DEGs. (A)List of GO terms with the top 30. (B) Top 20 significantly enriched downregulated KEGG pathways. (C) Top 20 significantly enriched upregulated KEGG pathways. (D) Levels of changes in genes associated with TG,PC,PE and CL metabolism, n = 3-4 per group. *P < 0.05, **P < 0.01, ***P < 0.001.

To identify the function of the identified DEGs, we performed GO and KEGG pathway enrichment analyses. The identified DEGs were primarily enriched in the lipid metabolic process and fatty acid metabolic process and the significantly enriched GO terms were mainly related to the biological process (Fig 8A). KEGG pathway enrichment analysis was used to demonstrate the significance of gene enrichment in different pathways, revealing that the DEGs were significantly enriched in 34 downregulated and 14 upregulated pathways (*P* < 0.05). Fig 8B and 8C shows the top 20 downregulated and upregulated KEGG enrichment pathways. The top 20 downregulated KEGG enrichment pathways includes the glycerolipid metabolism pathway, which regulates TG metabolism, and the glycerophospholipid metabolism pathway, which regulates PE, PC, and CL metabolism.

Eight genes *(Lipg*, *Gpat4*, *Lpin2*, *Dgkh*, *Pnpla2*, *Chkb*, *Etnppl*, *and Gpcpd1*) are involved in the glycerolipid and glycerophospholipid metabolic pathways. Notably, among the DEGs, the mRNA expression levels of *Gpat4*, *Gpcpd1*, *Chkb*, and *Etnppl* were downregulated in the MASLD group compared to those in the control group (Fig 8D). The protein encoded by the glycerol-3-phosphate acyltransferase (*Gpat4*) gene is related to TG metabolism, whereas those encoded by glycerophosphocholine phosphodiesterase 1 (*Gpcpd1*), choline kinase beta (*Chkb*), and ethanolamine phosphate phospholyase (*Etnppl*) genes are related to the metabolism of PC, PE, and CL.

### 3.8. Correlation analysis of differentially expressed lipid metabolites and DEGs

**Fig 9:**
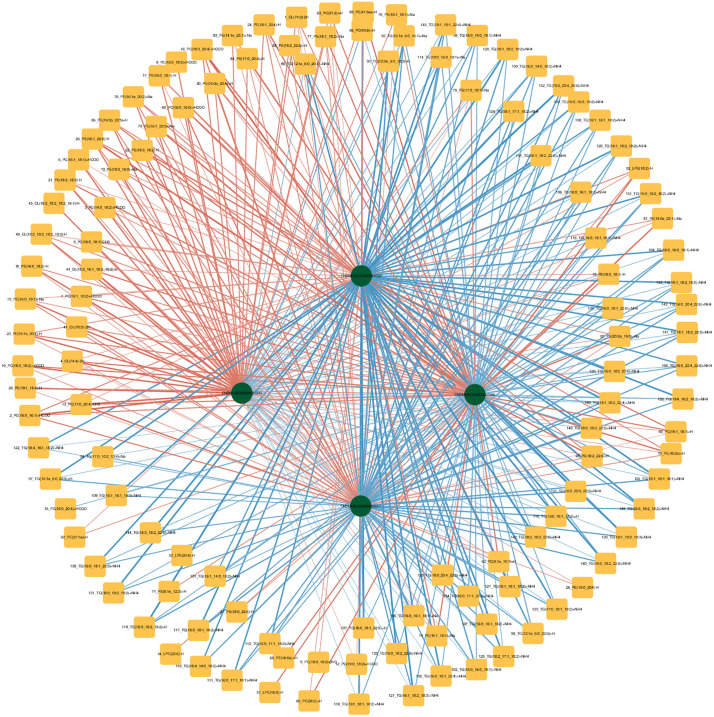
Differential lipid metabolites and differential gene networks. Squares represent lipid metabolites, circles represent differential genes, red represents positive correlation, blue represents negative correlation, and the darker the color, the greater the correlation.

Fig 9 shows the correlation analysis of the four DEGs *Gpat4*, *Gpcpd1*, *Chkb,* and *Etnppl* with the differentially expressed lipid metabolites TG, PE, PC, and CL.

## 4. Discussion

The results showed that a high-fat diet was associated with an increased risk of fatty liver disease and glucose intolerance in male mice [20,21]. Therefore, in the present study, we selected male mice to establish a high-fat diet-induced MASLD mode. HFD-induced MASLD model [22,23] was used to demonstrate that long-term HFD intake affects the metabolism of glycerolipids and glycerophospholipids. We found that the content of TG involved in glycerolipid metabolism increased significantly in the liver of MASLD-model mice, whereas that of PE, PC, and CL, involved in glycerophospholipid metabolism, decreased significantly.

Under normal circumstances, the liver stores very little TG. Rather, TG is collected by the liver following its absorption through the intestine, and enters the blood as a component of very low density lipoprotein (VLDL), hydrolyzed by lipoprotein lipase and liver triglyceride lipase, and eventually converted to low density lipoprotein (LDL) [24]. In the case of non-alcoholic fatty liver disease, excess TG storage leads to lipotoxicity. These observations are consistent with the results of the present study. In particular, the development of insulin resistance leads to the accumulation of TG in the liver owing to enhanced fatty acid entry into the liver and increased *de novo* lipogenesis [25]. In addition, higher fasting TG levels are observed in insulin resistance patients [26]. The current study revealed that insulin resistance was significantly increased in MASLD mice. Some studies have shown that fenofibrate can effectively improve liver steatosis and insulin resistance [27,28]. Studies have also shown that the new antidiabetic drug GLP-1RA may prevent or delay the development of MASLD by reducing fat accumulation in the liver [29]. Semiglutide can also mitigate hepatic steatosis by reducing the levels of lipogenic end products, and reduce lipotoxic fatty acids and lipase, thereby alleviating liver TG content and lipogenesis [30], benefiting patients with MASLD. Fatty acid flux is regulated through the glycerol synthesis pathway, in which GPAT activity is a rate-limiting step [31]. Moreover, abnormal lipid accumulation is associated with dyshomeostasis of endoplasmic reticulum proteins in hepatocytes [32]. Thus, considering that GPAT4 is located in the endoplasmic reticulum [31], the downregulation of *Gpat4* expression observed in the present study was likely related to the stress injury of the liver endoplasmic reticulum in MASLD.

PE and PC are essential for the structure and function of eukaryotic membranes, accounting for more than 50% of the total phospholipid species in eukaryotic biofilms [33]. PE has many biological functions such as oxidative phosphorylation, mitochondrial biogenesis and autophagy [34]. In particular, PE in the mitochondrial membranes of hepatocytes is involved in glucose metabolism and oxidative phosphorylation in mice [34]. In MASLD, excessive lipid accumulation in the liver leads to the production of lipotoxins, which in turn can lead to mitophagy, oxidative phosphorylation, and mitochondrial biogenesis [35]. Moreover, as PE is especially abundant in the inner membrane of mitochondria [36], decreased mitochondrial PE levels affect the activity of the respiratory chain, leading to significantly reduced ATP production [37]. In this study, lipidomic analyses indicated that PE content was significantly reduced and glucose metabolism was impaired in the liver of MASLD mice, which was consistent with the above findings. The ETNPPL protein is expressed in large quantities in the liver and brain, where it degrades phosphoethanolamine [38], a small amino acid that is involved in various lipid metabolic pathways and necessary for PE production [39]. Downregulation of *Etnppl* disrupts lipid synthesis in human hepatocellular carcinoma and cholangiocarcinoma, consistent with the results of gene set enrichment analysis suggesting that *Etnppl* is a regulator of lipogenesis [40]. In this study, the downregulation of *Etnppl* may indirectly affects the generation of PE, which may affect mitochondrial function in the liver and promote lipid accumulation. However, as current MASLD models are time-limited, future research into the effects of MASLD at different time points is warranted to evaluate long-term metabolism characteristics.

PC regulates cell signal transduction and is primarily synthesized in the liver. Decreased liver PC levels affect the integrity of the plasma membrane, allowing pro-inflammatory molecules to pass into liver cells, thereby causing molecular damage and promoting steatohepatitis development [41]. Consumption of PC can alter protein transport and lipid accumulation and may be associated with human NAFLD [42,43]. PC can change the polarization of macrophages and reduce the inflammatory response [44]. Studies have shown that PC supplementation can significantly reduce the development of fatty liver and inflammation [44]. Hence, The complementary treatment with PC provides a new strategy in the potential drug therapies for MASLD. Gpcpd 1 is a key enzyme in the metabolism of choline,which is a precursor of PC. Choline metabolism affects the clearance of TG in the liver. In this study, the content of PC and *Gpcpd 1* expression in the liver of MASLD mice was significantly decreased, which may indirectly affect the clearance of TG in the liver. In turn, *Chkb* encodes a rate-limiting enzyme for the synthesis of PC, which also constitutes a major mitochondrial membrane phospholipid [45]. Specifically, CHKB catalyzes the first step in *de novo* synthesis of PC and PE via the Kennedy pathway. Disturbance of this pathway may also affect mitochondrial membrane homeostasis [46]. In turn, we found that the expression of *Chkb* was decreased in MASLD mice, thereby affecting the synthesis of PC and PE.

Mitochondria are the only producers of CL in eukaryotic cells and produce CL on the stromal side of the mitochondrial inner membrane. CL is present as a phospholipid dimer in the inner membrane of the mitochondria and plays a key role in mitochondrial oxidative phosphorylation [47,48]. CL oxidation is associated with mitochondrial dysfunction in MASLD [49]. In the present study, the content of CL in the liver of MASLD mice was decreased, indicating that hepatic mitochondrial function was impaired, thereby affecting liver function. Moreover, HFD-induced fatty liver in mice is associated with a reduction in (18:2)4 CL [50], consistent with the results of lipidomics in the present study, which found significantly reduced (18:2)4 CL in the liver of MASLD. As the activity of PE and CL is closely related to mitochondrial function, drugs that improve mitochondrial function may also further improve liver lipid deposition. Currently, no approved drugs are available for the treatment of MASLD. The findings of this study provide a certain reference value for the research and development of MASLD-targeting drugs in the future.

## 5. Conclusions

In summary, using lipidomic and transcriptomic methods, we analyzed changes in lipid metabolism in HFD-induced MASLD mice. Our study demonstrated that glycerolipid and glycerophospholipid metabolic activities are altered in MASLD mice. This study provides important insights regarding the mechanisms associated with changes in lipid metabolism in HFD-induced MASLD.

## Supporting information

S1 File Supplemental Figures and Tables for the primers sequences of liver-related genes, liver ALT, liver AST and Lipidomics-related indicators. (DOCX)

S1 Data Lipidomics minimal dataset. (XLSX)

S2 Data Transcriptomic minimal dataset. (XLS)

S3 Data Minimum dataset for multi-omics integration analysis. (XLSX)

## Abbreviations

ALT: alanine transaminase
AST: glutamic-oxalacetic transaminase
CL: cardiolipin
*Chkb*: choline kinase beta
DEG: differentially expressed gene
*Etnppl*: ethanolamine phosphate phospholyase
FC: fold change
GO: Gene Ontology
*Gpat*: glycerol-3-phosphate acyltransferase
*Gpcpd1*: glycerophosphocholine phosphodiesterase 1
*Chkb*: Choline kinase beta
*Etnppl*: ethanolamine phosphate phospholyase
H&E: hematoxylin and eosin
HFD: high-fat diet
HOMA-IR: homeostatic model assessment of insulin resistance
KEGG: Kyoto Encyclopedia of Genes and Genome
LDL-C: low-density lipoprotein cholesterol
MASLD: metabolic-dysfunction-associated steatotic liver disease
NAFLD: non-alcoholic fatty liver disease
NEFA: non-esterified fatty acid
PC: phosphatidylcholine
PCA: principal component analysis
PE: phosphatidylethanolamine
PLS-DA: partial least squares discrimination analysis
RNA-Seq: RNA-sequencing
RT-qPCR: Reverse transcription quantitative polymerase chain reaction
TCH: total cholesterol
TG: triglycerides
VLDL: very low-density lipoprotein
