# Supplementary material for: Multi-omics analysis reveals metabolic regulation of phosphatidylcholine, triglycerides, phosphatidylethanolamine, and cardiolipin metabolism in mouse liver with metabolic dysfunction-associated steatotic liver disease
Source: PLoS One. 2025 Nov 7;20(11):e0332177. doi: 10.1371/journal.pone.0332177 (PMC12594369; doi:10.1371/journal.pone.0332177)
Supplement: S1 File — (DOCX) [file pone.0332177.s001.docx]

Supplementary Material

**Supplementary Table 1. Sequences of PCR primers of liver**

| Name | FORWARD(5’-3’) | REVERSE(5’-3’) | prodSize | GenBank accession number |
| --- | --- | --- | --- | --- |
| 18s | TTGACGGAAGGGCACCACCAG | GCACCACCACCCACGGAATCG | 116bp | NR_003278.3 |
| Gpat4 | TCCAGCGGCGTAGACAAAGAC | TGAGGCTCGGCTCCAGTCC | 115bp | NM_018743.4 |
| Gpcpd1 | GGATACCTGCCTTTCCCACACC | CCTGCCTGCTGCCACACC | 143bp | NM_001042672.1 |
| Chkb | AACCTGCTCTTCCGATGCTCAC | CTGCGAGAATGGCGAACATCAC | 145bp | NM_007692.6 |
| Etnppl | GACCACGAAGACCCATCCACTG | ATCTTCCTTCCGCTGCTGTGAG | 80bp | NM_001163587.1 |
| Pnpla2 | GCTGCTGTGGTGGAGGAGAG | TGGAAAGGGTGGTCATCAGGTC | 148bp | NM_001163689.1 |
| Lipg | CCCAGCCCACCCTCTACATTAC | GATCGCCCAAGTCCTCCTCAG | 115bp | NM_010720.3 |
| Dgkh | TCTTGGTTCAGGAGGTTGGAAGG | GAAACACATCACAGCAAACACACTC | 127bp | NM_001081336.2 |
| Lpin2 | GCTCCGTGAGTTACTTGTGTTAGG | TACTGTGGTGGTGATGGCTTAGG | 135bp | NM_001164885.1 |

##
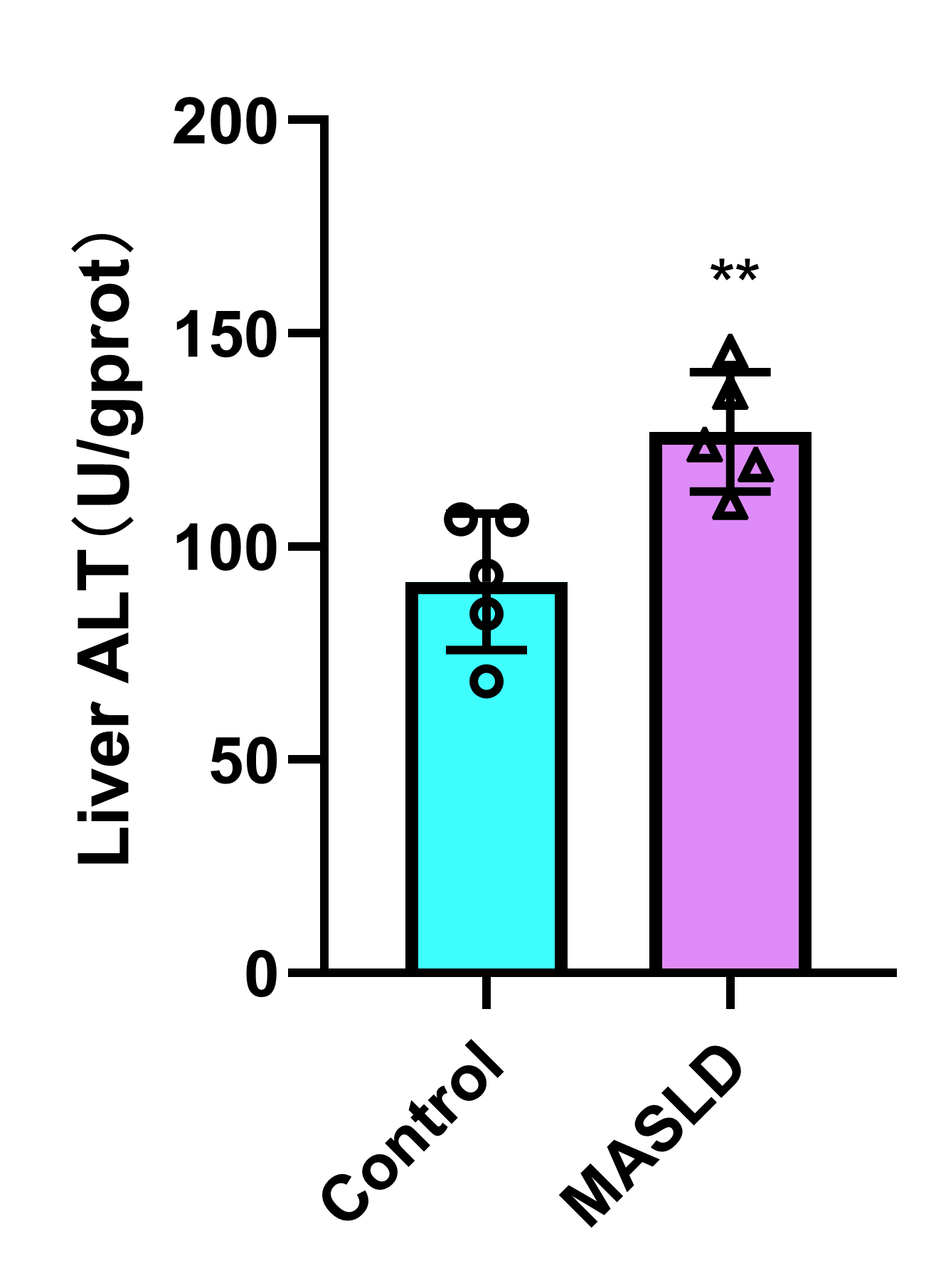


## **Supplementary Figure 1.** Liver tissue alanine aminotransferase (ALT)


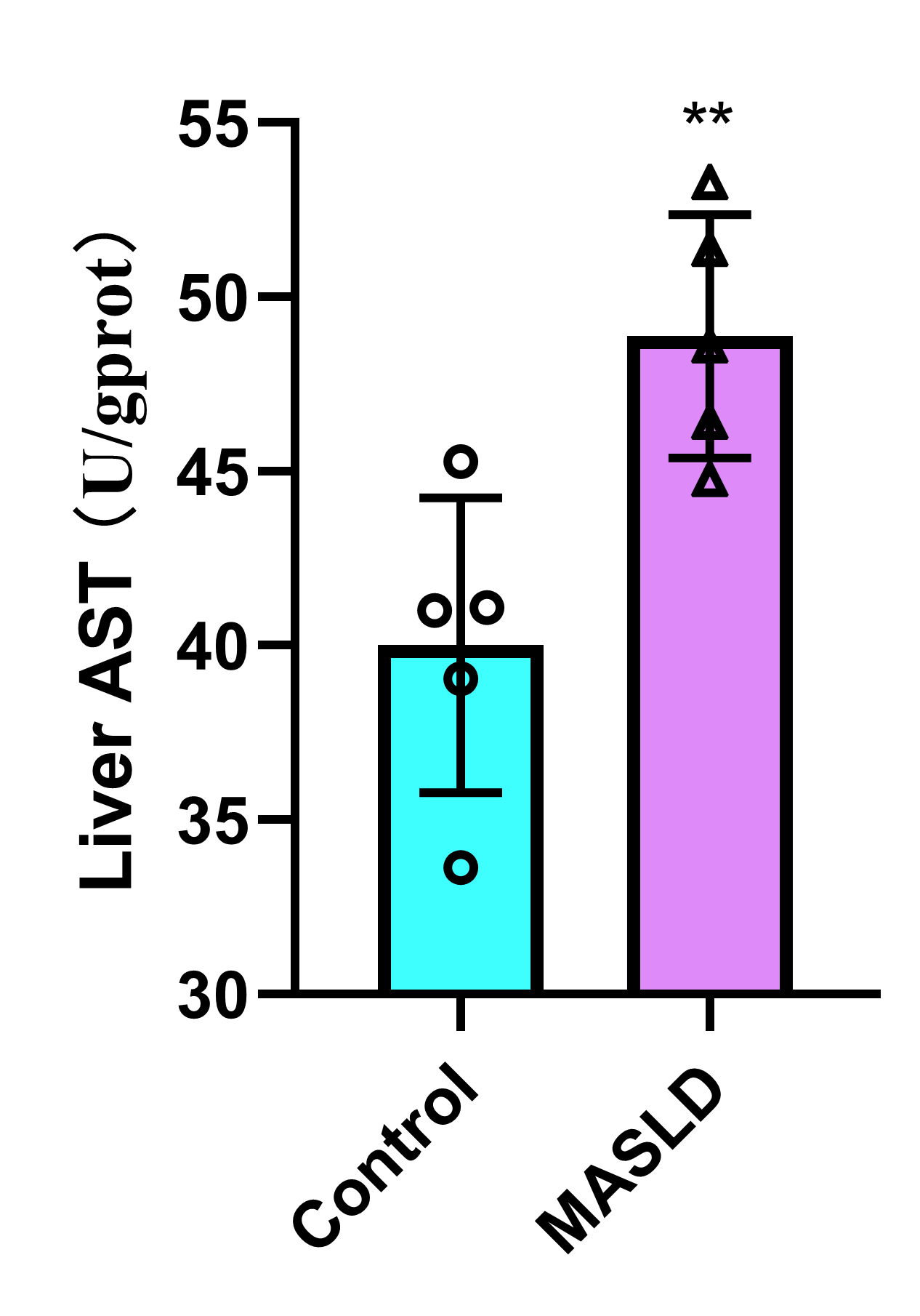


**Supplementary Figure 2.** Liver tissue aspartate aminotransferase (AST)

| Sample Group | A | R^2^X（cum） |
| --- | --- | --- |
| QC | 2 | 0.835 |
| Control VS MASLD | 2 | 0.84 |

**Supplementary Table 2** PCA Model Parameters between control group and MASLD group.

**Supplementary Table 3** PLS—DA Model evaluation Parameters between control group and MASLD group.

| Sample Group | A | R^2^X（cum） | R^2^Y（cum） | Q^2^（cum） |
| --- | --- | --- | --- | --- |
| Control VS MASLD | 3 | 0.86 | 0.995 | 0.935 |

**Supplementary Table 4** OPLS—DA Model evaluation Parameters between control group and MASLD group.

| Sample Group | A | R^2^X（cum） | R^2^Y（cum） | Q^2^（cum） |
| --- | --- | --- | --- | --- |
| Control VS MASLD | 1+2 | 0.86 | 0.995 | 0.925 |

**Supplementary Table 5** Differences in lipids (TG,PC, PE and CL) between control group and MASLD group.

| **name** | **Lipid Ion** | **Ion Formula** | **CalMz** | **RT-(min)** | **Fold Change** | **P-value** | **VIP** |
| --- | --- | --- | --- | --- | --- | --- | --- |
| POS3797 | TG(12:0e_6:0_18:3)+H | C39 H71 O5 | 619.5296015 | 14.70762556 | 5.733664457 | 0.003495039 | 1.529912068 |
| POS3770 | TG(12:1e_6:0_16:1)+Na | C37 H68 O5 Na1 | 615.4958965 | 12.833 | 3.696538337 | 0.008050545 | 1.949185291 |
| POS3845 | TG(12:1e_6:0_20:4)+NH4 | C41 H74 O5 N1 | 660.5561505 | 13.88446682 | 5.509984438 | 0.008282401 | 2.207830833 |
| POS3850 | TG(12:1e_6:0_20:5)+H | C41 H69 O5 | 641.5139515 | 12.89753612 | 3.382804036 | 0.017189912 | 2.089561992 |
| POS3884 | TG(12:1e_6:0_22:6)+H | C43 H71 O5 | 667.5296015 | 13.2293017 | 5.876487335 | 0.010033358 | 1.041816542 |
| POS4066 | TG(15:0_16:0_18:1)+NH4 | C52 H102 O6 N1 | 836.7701655 | 17.82783881 | 11.63471125 | 0.004145771 | 1.111381194 |
| POS4069 | TG(15:0_16:0_18:2)+NH4 | C52 H100 O6 N1 | 834.7545155 | 17.56904662 | 6.691357188 | 0.007372707 | 1.028767605 |
| POS4269 | TG(15:0_18:2_18:2)+NH4 | C54 H100 O6 N1 | 858.7545155 | 17.29911383 | 6.046924987 | 0.005541602 | 1.664920281 |
| POS4987 | TG(15:0_18:2_22:6)+NH4 | C58 H100 O6 N1 | 906.7545155 | 17.02373068 | 9.851092704 | 0.00945862 | 1.011898451 |
| POS4010 | TG(16:0_14:0_18:1)+NH4 | C51 H100 O6 N1 | 822.7545155 | 17.6620987 | 9.093992704 | 0.006623694 | 1.891018246 |
| POS4035 | TG(16:0_14:0_18:2)+NH4 | C51 H98 O6 N1 | 820.7388655 | 17.38903682 | 7.604090114 | 0.006383091 | 3.323272678 |
| POS4105 | TG(16:0_16:0_18:1)+NH4 | C53 H104 O6 N1 | 850.7858155 | 17.95604359 | 11.01702045 | 0.005775815 | 5.606795246 |
| POS4151 | TG(16:0_16:1_18:1)+Na | C53 H98 O6 Na1 | 853.7255615 | 17.1919867 | 5.708613856 | 0.006096898 | 1.541765857 |
| POS4153 | TG(16:0_16:1_18:1)+NH4 | C53 H102 O6 N1 | 848.7701655 | 17.69461823 | 9.249435419 | 0.005118056 | 6.853973648 |
| POS4185 | TG(16:0_16:1_18:2)+NH4 | C53 H100 O6 N1 | 846.7545155 | 17.40583302 | 8.419472742 | 0.005042866 | 8.917789469 |
| POS4250 | TG(16:0_17:1_18:1)+NH4 | C54 H104 O6 N1 | 862.7858155 | 17.84382357 | 14.87851873 | 0.002723431 | 3.267192671 |
| POS4261 | TG(16:0_17:1_18:2)+NH4 | C54 H102 O6 N1 | 860.7701655 | 17.57929666 | 8.80587755 | 0.003437232 | 2.718577299 |
| POS4981 | TG(16:0_17:1_22:6)+NH4 | C58 H102 O6 N1 | 908.7701655 | 17.32485861 | 13.71618213 | 0.001606654 | 1.125409751 |
| POS4344 | TG(16:0_18:1_18:1)+NH4 | C55 H106 O6 N1 | 876.8014655 | 17.9532218 | 14.88141977 | 0.009112304 | 17.0921943 |
| POS4423 | TG(16:0_18:1_18:2)+H | C55 H101 O6 | 857.7592665 | 17.72470092 | 5.33527037 | 0.005963201 | 1.583694161 |
| POS4427 | TG(16:0_18:1_18:2)+NH4 | C55 H104 O6 N1 | 874.7858155 | 17.88973421 | 6.730346258 | 0.007288327 | 14.7226284 |
| POS5094 | TG(16:0_18:1_22:4)+NH4 | C59 H108 O6 N1 | 926.8171155 | 17.82960417 | 8.085980551 | 0.003484251 | 5.345163475 |
| POS5125 | TG(16:0_18:1_22:5)+H | C59 H103 O6 | 907.7749165 | 17.17299128 | 8.545009239 | 0.006057232 | 1.345852242 |
| POS5129 | TG(16:0_18:1_22:5)+NH4 | C59 H106 O6 N1 | 924.8014655 | 17.61766715 | 9.252765546 | 0.0033791 | 6.935955515 |
| POS5167 | TG(16:0_18:1_22:6)+NH4 | C59 H104 O6 N1 | 922.7858155 | 17.35028103 | 4.333962602 | 0.005952958 | 3.621602537 |
| POS4496 | TG(16:0_18:2_18:2)+H | C55 H99 O6 | 855.7436165 | 17.66247667 | 5.094889077 | 0.007515076 | 2.027702711 |
| POS4872 | TG(16:0_18:2_20:4)+NH4 | C57 H102 O6 N1 | 896.7701655 | 17.60529807 | 12.37254523 | 0.007060475 | 2.715447285 |
| POS5205 | TG(16:0_18:2_22:6)+NH4 | C59 H102 O6 N1 | 920.7701655 | 17.16518087 | 10.52065937 | 0.007156993 | 7.635266641 |
| POS5293 | TG(16:0_20:4_22:6)+NH4 | C61 H102 O6 N1 | 944.7701655 | 17.13503403 | 10.1421103 | 0.010656546 | 2.53851987 |
| POS5475 | TG(16:0_22:4_22:6)+NH4 | C63 H106 O6 N1 | 972.8014655 | 17.35007923 | 8.567697451 | 0.009287053 | 1.566754907 |
| POS5482 | TG(16:0_22:5_22:6)+NH4 | C63 H104 O6 N1 | 970.7858155 | 17.10885742 | 9.693596661 | 0.006944044 | 1.89343089 |
| POS4047 | TG(16:1_14:0_18:2)+NH4 | C51 H96 O6 N1 | 818.7232155 | 17.05942425 | 4.415226824 | 0.015618735 | 1.8944577 |
| POS4212 | TG(16:1_16:1_18:2)+NH4 | C53 H98 O6 N1 | 844.7388655 | 17.10933765 | 6.388400199 | 0.006641216 | 4.247320517 |
| POS4226 | TG(16:1_16:1_18:3)+NH4 | C53 H96 O6 N1 | 842.7232155 | 16.80104239 | 4.10886602 | 0.014455649 | 1.875342959 |
| POS4493 | TG(16:1_18:1_18:2)+NH4 | C55 H102 O6 N1 | 872.7701655 | 17.46194289 | 6.168362227 | 0.005858193 | 11.93863368 |
| POS4540 | TG(16:1_18:2_18:2)+NH4 | C55 H100 O6 N1 | 870.7545155 | 17.17887725 | 5.943287196 | 0.004573584 | 11.3355672 |
| POS4572 | TG(16:1_18:2_18:3)+NH4 | C55 H98 O6 N1 | 868.7388655 | 16.9565391 | 9.865235779 | 0.005323592 | 4.814003177 |
| POS5232 | TG(16:1_18:2_22:6)+NH4 | C59 H100 O6 N1 | 918.7545155 | 16.84496183 | 9.184954488 | 0.011607176 | 5.019391878 |
| POS3826 | TG(17:0_10:2_10:4)+Na | C40 H64 O6 Na1 | 663.4595115 | 11.853 | 3.238724834 | 0.022861748 | 2.112397635 |
| POS4613 | TG(17:0_18:1_18:2)+NH4 | C56 H106 O6 N1 | 888.8014655 | 17.86327998 | 7.463180782 | 0.002395212 | 2.935732504 |
| POS4287 | TG(18:0_16:0_18:1)+Na | C55 H104 O6 Na1 | 883.7725115 | 17.77697254 | 9.270463004 | 0.002369733 | 1.29248865 |
| POS5410 | TG(18:0_18:2_22:6)+NH4 | C61 H106 O6 N1 | 948.8014655 | 17.67352972 | 12.84547781 | 0.0032284 | 4.106243901 |
| POS5537 | TG(18:0_20:4_22:5)+NH4 | C63 H108 O6 N1 | 974.8171155 | 17.39388635 | 10.05398816 | 0.007951158 | 1.012131192 |
| POS4623 | TG(18:1_17:1_18:2)+NH4 | C56 H104 O6 N1 | 886.7858155 | 17.59523059 | 5.588285971 | 0.006180559 | 1.864570052 |
| POS4734 | TG(18:1_18:1_18:1)+NH4 | C57 H108 O6 N1 | 902.8171155 | 17.98288671 | 8.347082985 | 0.00369344 | 7.586896314 |
| POS4782 | TG(18:1_18:1_18:2)+NH4 | C57 H106 O6 N1 | 900.8014655 | 17.76358244 | 5.89393159 | 0.005500457 | 7.712547143 |
| POS5369 | TG(18:1_18:1_22:4)+NH4 | C61 H110 O6 N1 | 952.8327655 | 17.88012986 | 8.523548602 | 0.003384257 | 2.598971428 |
| POS4828 | TG(18:1_18:2_18:2)+NH4 | C57 H104 O6 N1 | 898.7858155 | 17.55844294 | 4.849845839 | 0.008896903 | 5.679772913 |
| POS4871 | TG(18:1_18:2_18:3)+NH4 | C57 H102 O6 N1 | 896.7701655 | 17.32841432 | 6.451473804 | 0.006571459 | 6.893724691 |
| POS5388 | TG(18:1_18:2_22:4)+NH4 | C61 H108 O6 N1 | 950.8171155 | 17.59918627 | 7.053405419 | 0.005424104 | 2.680859373 |
| POS5408 | TG(18:1_18:2_22:5)+NH4 | C61 H106 O6 N1 | 948.8014655 | 17.47711575 | 12.03231758 | 0.003124242 | 4.153636403 |
| POS4633 | TG(18:2_17:1_18:2)+NH4 | C56 H102 O6 N1 | 884.7701655 | 17.30432626 | 4.956798494 | 0.005081901 | 1.220871077 |
| POS5292 | TG(18:2_18:2_22:6)+NH4 | C61 H102 O6 N1 | 944.7701655 | 16.91101262 | 5.084887584 | 0.037359732 | 2.987927293 |
| POS5488 | TG(18:2_20:4_22:6)+NH4 | C63 H102 O6 N1 | 968.7701655 | 16.83282293 | 5.773175254 | 0.03580902 | 1.621049862 |
| POS4902 | TG(18:3_18:2_18:2)+NH4 | C57 H100 O6 N1 | 894.7545155 | 16.99056975 | 4.921825499 | 0.015023561 | 5.911179004 |
| POS5308 | TG(18:3_18:2_22:6)+NH4 | C61 H100 O6 N1 | 942.7545155 | 16.58316865 | 6.739253187 | 0.043558274 | 1.894977908 |
| POS4240 | TG(18:4_14:0_18:2)+NH4 | C53 H94 O6 N1 | 840.7075655 | 16.66625817 | 6.443030524 | 0.004893963 | 1.239236572 |
| POS4586 | TG(18:4_16:1_18:2)+NH4 | C55 H96 O6 N1 | 866.7232155 | 16.62251899 | 4.890748974 | 0.022691695 | 2.039893608 |
| POS4930 | TG(18:4_18:2_18:2)+NH4 | C57 H98 O6 N1 | 892.7388655 | 16.76642018 | 6.545726346 | 0.012987523 | 3.355856072 |
| POS4975 | TG(19:1_18:2_18:3)+NH4 | C58 H104 O6 N1 | 910.7858155 | 17.52802146 | 9.466948551 | 0.003005706 | 1.107399966 |
| POS5431 | TG(22:5_18:2_18:2)+NH4 | C61 H104 O6 N1 | 946.7858155 | 16.91773241 | 6.690626193 | 0.046665594 | 1.524780919 |
| NEG46 | CL(18:2_16:1_18:2_18:2)-H | C79 H139 O17 P2 | 1421.949305 | 16.26147999 | 0.36994976 | 0.000531717 | 1.234539715 |
| NEG48 | CL(18:2_18:2_18:2_16:1)-H | C79 H139 O17 P2 | 1421.949305 | 16.762834 | 0.309687194 | 0.035053519 | 1.250985349 |
| NEG72 | CL(18:2_18:2_18:2_18:2)-H | C81 H141 O17 P2 | 1447.964955 | 16.34385933 | 0.738126376 | 0.015156494 | 2.761876734 |
| NEG39 | CL(70:3)-2H | C79 H146 O17 P2 | 714.502314 | 10.837 | 0.417429181 | 0.00261849 | 3.53545637 |
| NEG100 | CL(74:2)-2H | C83 H156 O17 P2 | 743.541439 | 10.45478695 | 0.681991764 | 0.018089489 | 1.316049135 |
| NEG102 | CL(74:4)-2H | C83 H152 O17 P2 | 741.525789 | 10.848 | 0.586862282 | 0.006533798 | 1.783418757 |
| NEG1008 | PC(14:0_18:2)+HCOO | PC(14:0_18:2)+HCOO | 774.5290605 | 9.191842918 | 0.554818478 | 0.000820256 | 1.254488217 |
| NEG994 | PC(16:0_16:0)+HCOO | PC(16:0_16:0)+HCOO | 778.5603605 | 11.26471636 | 0.628517254 | 0.000369013 | 3.148299767 |
| NEG1003 | PC(16:0_16:1)+HCOO | C41 H79 O10 N1 P1 | 776.5447105 | 10.17806122 | 0.60400679 | 0.006259686 | 1.595477261 |
| NEG1035 | PC(16:0_18:1)-CH3 | C41 H79 O8 N1 P1 | 744.5548805 | 12.9013927 | 0.723857878 | 0.004070346 | 1.710528119 |
| NEG1079 | PC(16:0_18:3)+HCOO | C43 H79 O10 N1 P1 | 800.5447105 | 9.829463027 | 0.607895192 | 0.018571902 | 2.397112094 |
| NEG1182 | PC(16:0_20:5)+HCOO | C45 H79 O10 N1 P1 | 824.5447105 | 9.685085767 | 1.219847478 | 0.038351718 | 1.054059726 |
| NEG1048 | PC(16:1_18:1)+HCOO | C43 H81 O10 N1 P1 | 802.5603605 | 10.45261988 | 0.734974894 | 0.032220344 | 8.822193691 |
| NEG1077 | PC(16:1_18:2)+HCOO | C43 H79 O10 N1 P1 | 800.5447105 | 9.360969269 | 0.476527924 | 0.00077363 | 3.224394691 |
| NEG1215 | PC(17:0_20:4)-CH3 | C44 H79 O8 N1 P1 | 780.5548805 | 12.20606187 | 0.299906168 | 0.001266213 | 1.425364268 |
| NEG1132 | PC(18:0_18:2)-CH3 | C43 H81 O8 N1 P1 | 770.5705305 | 13.11893477 | 0.653186991 | 0.030021461 | 1.241955819 |
| NEG1236 | PC(18:0_20:3)+HCOO | C47 H87 O10 N1 P1 | 856.6073105 | 11.9584808 | 2.212422248 | 0.033774145 | 1.461761847 |
| NEG1242 | PC(18:0_20:4)+HCOO | C47 H85 O10 N1 P1 | 854.5916605 | 11.4748572 | 1.27472567 | 0.019714413 | 4.1330064 |
| NEG1178 | PC(18:3_18:2)+HCOO | C45 H79 O10 N1 P1 | 824.5447105 | 8.795470775 | 0.278437258 | 0.000193247 | 1.700026948 |
| NEG1295 | PC(18:3_20:4)+HCOO | C47 H79 O10 N1 P1 | 848.5447105 | 8.700722705 | 0.64396673 | 0.002024572 | 1.949155705 |
| NEG1207 | PC(19:0_18:2)+HCOO | C46 H87 O10 N1 P1 | 844.6073105 | 12.23307223 | 0.460876171 | 0.016196414 | 1.367416772 |
| POS2435 | PC(19:1_18:1)+H | C45 H87 O8 N1 P1 | 800.6163835 | 12.43603216 | 0.590472613 | 0.016218783 | 1.350619046 |
| POS2020 | PC(31:2)+H | C39 H75 O8 N1 P1 | 716.5224835 | 10.89422109 | 0.387333235 | 0.00011724 | 1.57756083 |
| POS2698 | PC(40:6)+H | C48 H85 O8 N1 P1 | 834.6007335 | 11.23440411 | 1.417281455 | 0.006858219 | 2.144633774 |
| POS1971 | PC(8:1e_10:1)+H | C26 H51 O7 N1 P1 | 520.3397685 | 1.682459572 | 0.484364406 | 0.046459982 | 2.422656107 |
| POS3083 | PE(14:0e_23:1)+Na | C42 H84 O7 N1 P1 Na1 | 768.5877635 | 11.074 | 0.560952182 | 0.02226123 | 2.238373394 |
| POS3092 | PE(14:1e_23:1)+Na | C42 H82 O7 N1 P1 Na1 | 766.5721135 | 10.89996714 | 0.655959612 | 0.03556693 | 1.508658098 |
| POS2927 | PE(16:0_18:1)+Na | C39 H76 O8 N1 P1 Na1 | 740.5200785 | 10.0363399 | 0.134871608 | 0.002361804 | 1.43033302 |
| NEG1416 | PE(16:0_18:1)-H | C39 H75 O8 N1 P1 | 716.5235805 | 11.73752176 | 0.542758962 | 0.009567078 | 1.310241905 |
| NEG1420 | PE(16:0_18:2)-H | C39 H73 O8 N1 P1 | 714.5079305 | 10.77016483 | 0.401098185 | 0.002327182 | 5.409634708 |
| POS2821 | PE(16:0e)+H | C21 H45 O7 N1 P1 | 454.2928185 | 2.251547634 | 0.51058824 | 0.017953023 | 2.521032665 |
| POS3069 | PE(16:0p_20:4)+H | C41 H75 O7 N1 P1 | 724.5275685 | 11.27238901 | 0.63647301 | 0.013261853 | 1.797729061 |
| POS3191 | PE(16:0p_22:5)+H | C43 H77 O7 N1 P1 | 750.5432185 | 11.36334788 | 0.669689673 | 0.030128716 | 1.113550933 |
| POS2937 | PE(16:1_18:1)+Na | C39 H74 O8 N1 P1 Na1 | 738.5044285 | 9.584992349 | 0.482389367 | 0.028499225 | 1.3937019 |
| POS3059 | PE(16:1_20:3)+Na | C41 H74 O8 N1 P1 Na1 | 762.5044285 | 9.254907329 | 0.390508268 | 0.003735557 | 1.582960819 |
| NEG1541 | PE(16:1_22:6)-H | C43 H71 O8 N1 P1 | 760.4922805 | 9.146701715 | 0.558444902 | 0.002638075 | 1.300655404 |
| POS3053 | PE(16:1e_20:2)+Na | C41 H78 O7 N1 P1 Na1 | 750.5408135 | 11.37605048 | 0.672507064 | 0.033914513 | 1.107300636 |
| NEG1469 | PE(16:1e_20:4)-H | C41 H73 O7 N1 P1 | 722.5130155 | 11.18730096 | 0.631307643 | 0.010885367 | 1.531559169 |
| POS2962 | PE(17:0_18:1)+Na | C40 H78 O8 N1 P1 Na1 | 754.5357285 | 11.302 | 0.645060738 | 0.024349618 | 1.223513483 |
| POS3100 | PE(17:0_20:4)+H | C42 H77 O8 N1 P1 | 754.5381335 | 11.30620475 | 0.614457893 | 0.013734533 | 1.24636351 |
| POS2921 | PE(18:0_16:0)+Na | C39 H78 O8 N1 P1 Na1 | 742.5357285 | 11.01297618 | 0.500848273 | 0.004765926 | 4.590021928 |
| NEG1436 | PE(18:0_18:1)-H | C41 H79 O8 N1 P1 | 744.5548805 | 12.87284428 | 0.759651439 | 0.013748741 | 1.331361717 |
| NEG1567 | PE(18:0_22:5)-H | C45 H79 O8 N1 P1 | 792.5548805 | 11.8791795 | 0.650629639 | 0.002799976 | 1.0172001 |
| POS3021 | PE(18:1_18:1)+Na | C41 H78 O8 N1 P1 Na1 | 766.5357285 | 10.80070373 | 0.705065911 | 0.039024423 | 4.756180784 |
| POS3045 | PE(18:1_18:2)+Na | C41 H76 O8 N1 P1 Na1 | 764.5200785 | 9.845175594 | 0.470848299 | 0.02544974 | 1.798739606 |
| NEG1448 | PE(18:1_18:2)-H | C41 H75 O8 N1 P1 | 740.5235805 | 10.91135529 | 0.569370697 | 0.003981581 | 2.958544926 |
| NEG1514 | PE(18:1_20:4)-H | C43 H75 O8 N1 P1 | 764.5235805 | 10.69251654 | 0.703722462 | 0.022606401 | 3.949586599 |
| NEG1453 | PE(18:2_18:2)-H | C41 H73 O8 N1 P1 | 738.5079305 | 9.961463236 | 0.171404369 | 0.000594468 | 1.703253073 |
| POS3268 | PE(18:2_22:6)+H | C45 H75 O8 N1 P1 | 788.5224835 | 9.626961319 | 0.411056683 | 0.006607838 | 1.629312956 |
| NEG1591 | PE(18:2_22:6)-H | C45 H73 O8 N1 P1 | 786.5079305 | 9.428854215 | 0.429943087 | 0.001957953 | 1.591109045 |
| POS2862 | PE(18:2e)+H | C23 H45 O7 N1 P1 | 478.2928185 | 1.791493441 | 0.310439824 | 0.005896711 | 1.530119043 |
| NEG1467 | PE(18:3_18:2)-H | C41 H71 O8 N1 P1 | 736.4922805 | 9.443524773 | 0.498671834 | 0.008080667 | 1.207533143 |
| NEG1550 | PE(19:0_20:4)-H | C44 H79 O8 N1 P1 | 780.5548805 | 12.19086787 | 0.326553974 | 0.003748452 | 1.418844674 |
| POS3212 | PE(20:2e_19:0)+Na | C44 H86 O7 N1 P1 Na1 | 794.6034135 | 11.16262391 | 0.637544536 | 0.020888015 | 2.343483764 |
| POS3085 | PE(37:1e)+H | C42 H85 O7 N1 P1 | 746.6058185 | 12.2126699 | 0.588849307 | 0.032519877 | 2.540880083 |
| POS3111 | PE(38:2)+H | C43 H83 O8 N1 P1 | 772.5850835 | 12.35053038 | 0.632171568 | 0.030733446 | 1.600500542 |
| POS3275 | PE(41:5e)+H | C46 H85 O7 N1 P1 | 794.6058185 | 11.1539032 | 0.650279492 | 0.020142603 | 2.340387612 |
| POS2884 | PE(8:1e_12:3)+H | C25 H45 O7 N1 P1 | 502.2928185 | 2.581468064 | 0.665628824 | 0.021606165 | 1.01858152 |
